# Impact of 2017 ACC/AHA guideline on prevalence, awareness, treatment, control, and determinants of hypertension: a population-based cross-sectional study in southwest of Iran

**DOI:** 10.1186/s12963-021-00260-5

**Published:** 2021-05-25

**Authors:** Fatemeh Sadeghi, Bahman Cheraghian, Zahra Mohammadi, Sadaf G. Sepanlou, Sahar Masoudi, Zahra Rahimi, Leila Danehchin, Yousef Paridar, Farhad Abolnezhadian, Mohammad Noori, Seyed Ali Mard, Ali Akbar Shayesteh, Hossein Poustchi

**Affiliations:** 1 Department of Global Public Health, Karolinska Institute, Stockholm, Sweden; 2 Alimentary Tract Research Center, Imam Khomeini Hospital Clinical Research Development Unit, Department of Biostatistics and Epidemiology, School of Public Health, Ahvaz Jundishapur University of Medical Sciences, Ahvaz, Iran; 3 Liver and Pancreatobiliary Diseases Research Center, Digestive Diseases Research Institute, Tehran University of Medical Sciences, Tehran, Iran; 4 Digestive Disease Research Center, Digestive Disease Research Institute, Tehran University of Medical Sciences, Tehran, Iran; 5 Hearing Research Center, Department of Biostatistics and Epidemiology, School of Public Health, Ahvaz Jundishapur University of Medical Sciences, Ahvaz, Iran; 6 Behbahan Faculty of Medical Sciences, Behbahan, Iran; 7 School of medicine, Dezful University of Medical Sciences, Dezful, Iran; 8 Shoshtar Faculty of Medical Sciences, Shoshtar, Iran; 9 Ahvaz Jundishapur University of Medical Sciences, Ahvaz, Iran; 10 Abadan Faculty of Medical Sciences, Abadan, Iran; 11 Alimentary Tract Research Center, Imam khomeini Hospital Clinical Research Development Unit, School of Medicine, Ahvaz Jundishapur University of Medical Sciences, Ahvaz, Iran

**Keywords:** Hypertension, Iran, 2017 ACC/AHA hypertension guideline, JNC8 hypertension guideline, Risk factors, Prevalence, Awareness, Treatment, Control

## Abstract

**Background:**

In 2017, the American College of Cardiology/American Heart Association (ACC/AHA) provided a new guideline for hypertension prevention and management. We aimed to update the prevalence, awareness, control, and determinants of hypertension based on this guideline in Khuzestan province, southwest of Iran, and to estimate the number of people who are eligible for non-pharmacologic and pharmacologic intervention.

**Methods:**

This population-based cross-sectional study was conducted in Khuzestan, a large province in the southwest of Iran. Comprehensive information about the potential relating factors of hypertension was collected, blood pressure was measured, and anthropometric measurements were obtained. Moreover, the dietary pattern was evaluated in 2830 individuals, using a qualitative food frequency questionnaire.

**Results:**

Among 30,506 participants, 30,424 individuals aged 20–65 years were eligible for the study. In comparison with the previous guideline released by the Joint National Committee (JNC8), the prevalence of hypertension in Khuzestan dramatically increased from 15.81 to 42.85% after implementation of the ACC/AHA guideline, which was more dominant in the male population and the 45–54 age group. The sex and age adjustment of the hypertension prevalence was estimated to be 39.40%. The percentage of hypertension awareness, treatment, and control were 45.85%, 35.42%, and 59.63%, which dropped to 22.72%, 26.37%, and 28.94% after implementation of new guideline, respectively.

**Conclusions:**

In the ACC/AHA guideline, a higher number of individuals with the pre-hypertension condition were shifted into the hypertension category and the level of awareness, treatment, and control were dramatically decreased, which highlight a great need to expand the public health infrastructure for further managing the substantial increased burden on healthcare system. However, further studies with population over 65 years are required to estimate the eligibility for antihypertensive treatment in this province after implementation of new guideline.

## Background

Hypertension (HTN) is a major risk factor for non-communicable diseases such as cardiovascular diseases (CVD), stroke, and renal dysfunction. With 1.5 billion hypertensive people and 7.6 million HTN-related death, this disease remains major public health globally [1, 2]. In low- and middle-income countries (LMICs), such as Iran, the prevalence of HTN is increasing rapidly and it is estimated about three-fourths of the world’s hypertensive population will be from these countries by 2025 [2]. In Khuzestan province, a large proportion of the population is living with HTN [3]. Considering the high prevalence of HTN and its cost comorbidities, mainly on low resource settings with weak health systems, being updated about the indicators of HTN is essential to improve the approaches to control this disorder, and measuring progress towards the goals of Universal Health Coverage (UHC) of HTN.

The 6th 5-year National Plan of Economic, Social and Cultural Development of Islamic Republic of Iran (2017–2021) prioritizes the health of the population by expanding health service coverage and increasing financial protection mechanisms [4]. Achieving and sustaining UHC of HTN requires assessing the longer-term costs of health care, which is highly dependent on the HTN indicators such as prevalence, awareness, treatment, and control. According to the eighth report of the joint national committee (JNC8) guideline, patients with systolic blood pressure (SBP)≥140 mmHg and/or diastolic blood pressure (DBP) ≥90 mmHg were diagnosed with HTN [5]. Using this report, the overall prevalence, awareness, treatment and control of HTN in Iran were estimated to be 29.9%, 59.2%, 80.2%, and 39.1%, respectively [6]. However, recently the 2017 American College of Cardiology (ACC)/American Heart Association (AHA) recommended SBP/DBP≥130/80 mmHg as a new cut-off for HTN, indicating the HTN estimations are required to be updated in all regions for development of more cost-effective UHC interventions in the management of HTN [7]. To date, few studies have evaluated the impact of suggested guideline and have reported that shifting from JNC8 to 2017 ACC/AHA causes a significant increase in the prevalence of HTN and a drastic decline in awareness, treatment, and control of this disorder [6, 8, 9]. This impact is more dominated in LMICs like 15–49-year-old Indian people (from 13.0 to 40.6%) [9], which may impose a considerable impact on health systems due to the challenges in terms of coverage and access to health service.

HTN is a multifactorial disease, and several genetic, environmental, and behavioral factors contribute to its manifestation [10]. Low physical activity and overweight, as well as unhealthy diet such as high salt intake, are the leading risk factors of HTN [11]; nevertheless, considerable variations between countries and even within the countries exist. Although this disease is usually asymptomatic and people are not aware of their condition, it is one of the most preventable disorders and modifications of the lifestyle could considerably prevent the future progression of HTN into CVD and renal diseases. In the ACC/AHA guideline, a higher number of individuals with the pre-hypertension condition are shifted into the HTN category [7]. Being updated about the major determinants of HTN is a prerequisite for effective prevention and promotion of awareness strategies in the future.

Khuzestan is located in the of the southwest of Iran. This province with 4,936,000 population has been exposed to different life-threatening issues such as dust storms, extremely high temperatures, air pollution, and soil contamination [12]. According to JNC8 report, the prevalence of hypertension in Ahvaz, the capital city of this province, was estimated to be 17.58%, with 46.4% awareness and 22% control rate [3]. Among thirty-one provinces of Iran, this province had the lowest level of healthcare development and highest inequality of health services utilization in the country [13]. Therefore, the implementation of the 2017 ACC/AHA guideline may considerably delay the progression towards UHC of HTN in this province. Here, we aimed to update the estimates in a large sample of participants in Khuzestan Comprehensive Health Study to aid public health resources planning and prevention strategies.

## Methods

### Study design and study population

This cross-sectional health survey was conducted based on the data of Khuzestan Comprehensive Health Study (KCHS). KCHS is a large population-based cross-section study with the purpose of evaluating the health status of the Iranian adults in Khuzestan. This study was performed in the period of October 2016 to November 2018 and the participants were selected using a multistage random sampling method. In the first stage of cluster sampling, the health centers and health houses within each county were selected randomly (total 29 counties). According to the population of each county, a total number of 1079 of clusters, consisting of 780 health centers in urban and 299 health houses in rural areas, were chosen. Afterward, 30 individuals between 20 and 66 years old were selected within each cluster, using systematic random sampling.

The individuals who had been selected in the sampling process and were residents of Khuzestan province for at least 1 year were invited to participate in the study by a trained staff. We excluded individuals with mental, psychological or physical disabilities, or if they were reluctant to participate at any stage of the study. A written informed consent was obtained from all contributors before including them in the study. The sampling frame and study design has been described previously [14].

### Blood pressure and anthropometric measurements

The SBP and DBP were measured twice with 10-min interval in a seated position using analogue Riester sphygmomanometers. In addition, the participants’ height and weight were measured by Seca 206 body meter measuring tape and Seca 762 mechanical flat scale, respectively.

### Laboratory measurements

After overnight fasting, 15-ml peripheral blood was collected from each participant and was transferred to a research lab in a 4°C cold boxes within 3 h. After separating the serum, the levels of fasting blood sugar (FBS), total cholesterol, high-density lipoproteins (HDL) cholesterol, and low-density lipoprotein (LDL) cholesterol were measured.

### Dietary intake assessment

The dietary habits were assessed for 2830 randomly selected participants, and the consumption of sodium, total fat, fiber, and carbohydrate was measured per week. Briefly, an 86-item qualitative food frequency questionnaire (FFQ) was designed based on previous studies on major sources of the nutrients of interest in the Iranian diet. To estimate average food consumption frequency in the last year, we used closed-ended responses consisting of 9 categories: never or less than once/month, 1–3/month, 1–2/week, 3–4/week, 5–6/week, 1/day, 2–3/day, or >4/day. The standard portion size was estimated and the micronutrient and macronutrient information were obtained from the United States Department of Agriculture (USDA) database [15].

### Outcome definitions

According to the 2017 ACC/AHA guideline, HTN was defined if the SBP level was ≥130 mmHg and/or the DBP level was ≥80 mmHg and/or the participant takes antihypertensive medication [7]. These thresholds were SBP≥140 and/or DBP≥90 based on the JNC8 report [5].

Awareness was estimated among HTN-identified individuals if they responded positive to the self-reported of physician-diagnosed HTN. Treatment was defined as positive if self-aware individuals answered yes to the questions “Are you currently taking antihypertensive drug?” Moreover, the HTN control was defined among treated individuals with blood pressure <130/80 mmHg based on the 2017 ACC/AHA or <90/140 mmHg based on the JNC8 after taking anti-HTN drugs.

### Covariates definitions

Covariates included demographic, economic status, lifestyle, and CVD risk factors. Age groups included <25, 25–34, 35–44, 45–54, 55–64, or 65 years. Marital status consisted of single, married, divorced, or widow. Education was categorized according to the educational system in Iran as either illiterate (<1-year study), elementary school (1–5-year study), guidance school (6–8-year study), high school (9–12-year study), or universities (>12-year study) [16], and ethnicity classification was done based on major ethnic groups located in this province (Fars, Arab, Bakhtiary, Lor) [12]. The economic status was defined according to the wealth index, which was calculated by multiple correspondence analysis (MCA) with household’s ownership of selected assets and was further presented in quartiles [17].

Body mass index (BMI) was calculated by dividing weight (in kilograms) to height (in meters, squared) and further classified based on the WHO international BMI classification, as either underweight (<18.5 kg/m^2^), normal (18.5–24.9 kg/m^2^), overweight (25.0–29.9 kg/m^2^), or obesity (≥30) [18]. Waist-to-hip ratio (WHR) was the ratio of waist circumference to hip circumference. Physical activity was estimated by metabolic equivalent of task (MET) category, which was calculated based on the international physical activity questionnaire (IPAQ) guideline and classified as either insufficient (MET<600/week), or sufficient (MET ≥ 600/week) physical activity [19].

The status of smoking, drinking alcohol, and smoking water pipe were documented as either no, current, or former. These variables were recorded as current if the participant answered yes to “do you currently smoke, drink alcohol or smoke water pipe, regardless of the duration or frequency of consumption” OR recorded as former if the participant answered no to the last question but yes to “Did you use to smoke, drink alcohol, or smoke water pipe in the past and had quitted?”

Diabetes mellitus (DM) was defined as fasting plasma glucose levels (FBS) > 126 mg/dL, previously diagnosed DM by physician, or current intake of antidiabetic agents [20]. We documented heart disease if the participant reported any history of myocardial infarction, angiography, or any other related heart diseases. The positive family history of HTN was considered if the participant reported a history of HTN in the first-degree relatives. Dyslipidemia (DLP) was defined as either serum total cholesterol ≥ 200 mg/dL, HDL cholesterol <35 mg/dL, LDL cholesterol ≥130 mg/dL, or use of lipid-lowering medication [21].

### Statistical analysis

Multiple logistic regression was performed to identify the independent factors associated with prevalence, awareness, treatment, and control of HTN based on 2017 ACC/AHA guideline in the studied population. The final model was adjusted with established risk factors of HTN such as sex, age, BMI, and physical activity. The graphs were presented by GraphPad Prism 7 (GraphPad Prism Software, San Diego, CA, USA) and the statistical analysis was performed using STATA/SE version 12.0 software (STATA Corp., TX, USA). p-value <0.05 was considered as statistically significant.

## Results

### HTN indicators: 2017 ACC/AHA versus JNC8 hypertension guidelines

A total number of 30,506 individuals aged 20–65 years participated in this study. We excluded 82 subjects with missing information on SBP or DBP and included 30,424 participants in the final analysis. The mean (±SD) age was 41.71 (±11.89), and 64.26% of the participants were female.

According to JNC8 report, the overall prevalence of HTN in the studied population was 15.81%, which considerably increased to 42.85% after adopting the 2017 ACC/AHA guideline (Table 1). This difference was more dominant in male (17.79% vs 50.3%) and individuals between 45 and 54 years old (22.28 to 52.73%). Although 3.36% of the <25 years old were diagnosed with HTN based on JNC8, this level increased to 24.44% by applying the new guideline.

**Table 1:** Prevalence of hypertension and percentage of hypertension awareness, treatment, and control in Khuzestan province: 2017 ACC/AHA guideline vs NCJ8 report

Determinants of hypertension	Prevalence		Awareness		Treatment		Control	
	2017 ACC/AHA	JNC88	2017 ACC/AHA	JNC8	2017 ACC/AHA	JNC8	2017 ACC/AHA	JNC8
**Overall**	42.85	15.81	22.72	45.85	26.37	35.42	28.94	59.63
**Mean age (±SD)**	45.72 (11.71)	50.4 (10.17)	53.45 (8.11)	53.75 (7.89)	54.36 (7.51)	54.36 (7.51)	51.91 (9.72)	53.06 (8.84)
**Age group**								
<25	24.44	3.36	1.94	7.04	20.0	40.00	66.67	83.33
25–34	28.53	4.81	3.18	12.13	17.14	26.67	66.67	83.33
35–44	36.95	9.95	11.45	29.23	20.65	30.04	36.84	67.37
45–54	52.73	22.28	27.49	48.54	25.10	33.65	29.31	57.93
55–64	65.32	35.52	41.20	57.68	29.25	38.43	25.26	58.32
=65	75.45	45.51	53.17	68.42	22.39	28.85	13.33	40.00
**Gender**								
Male	50.30	17.79	14.90	31.06	24.91	33.78	26.51	54.62
Female	38.70	14.70	28.38	55.81	26.92	36.03	29.85	61.49
**Marital status**								
Single	31.02	5.75	4.61	16.27	23.08	35.29	50.00	75.00
Married	43.89	16.56	23.16	45.68	26.02	34.96	29.17	59.62
Divorced	38.66	12.89	21.74	43.48	10.00	15.00	50.00	75.00
Widowed	58.38	31.57	45.63	64.69	31.31	40.83	22.73	56.36
**Socioeconomic status**								
Q1	42.67	16.01	22.06	44.47	26.88	35.54	27.39	55.22
Q2	43.99	16.27	22.92	46.23	26.46	35.46	29.78	60.44
Q3	41.55	14.90	22.30	46.63	27.54	36.72	27.60	58.40
Q4	43.39	16.23	23.85	46.46	24.79	34.02	31.46	65.26
**Education**								
Illiterate	54.40	26.58	35.00	56.10	25.54	32.62	24.83	52.68
Elementary school	45.43	16.85	22.39	45.08	25.55	34.20	26.19	59.92
Guidance school	40.87	14.41	20.73	41.93	24.87	34.89	35.83	64.17
High school	38.02	11.65	16.10	37.15	29.11	41.18	33.15	67.39
Universities	33.29	8.55	13.65	35.95	30.68	45.38	33.85	60.00
**Ethnicity**								
Fars	45.63	18.08	24.24	46.05	39.26	52.15	35.64	70.24
Arab	44.68	15.46	21.47	44.64	13.37	18.58	16.74	45.58
Bakhtiary	36.79	14.13	24.14	47.68	33.17	43.71	31.65	59.92
Lor	42.84	17.68	23.28	46.71	50.52	61.00	28.98	59.09
Others	36.73	8.85	21.69	50.00	5.56	10.00	100.00	100.00
**BMI**								
Normal (18.6–25)	32.98	9.60	13.31	32.25	28.68	40.65	37.84	68.92
Underweight (≤18.5)	22.17	4.75	6.04	25.64	36.36	40.00	62.50	87.50
Overweight (25.1–30)	44.00	16.09	22.09	44.99	25.68	34.46	30.09	60.47
Obese (>30)	53.00	22.56	29.72	52.60	26.20	34.78	24.23	55.34
**Waist-hip ratio (WHR)**								
WHR<0.85	27.63	6.29	12.12	38.83	28.47	39.00	37.96	71.30
0.85≤ WHR <0.9	38.64	11.70	17.24	42.39	25.06	33.66	36.80	69.60
0.90≤ WHR <0.95	47.33	17.15	21.80	43.74	26.62	36.62	27.47	61.80
WHR ≥0.95	56.42	26.53	30.81	49.53	26.27	34.76	25.33	53.11
**Physical activity**								
Insufficient (MET<600/week)	51.81	21.63	24.71	45.49	25.48	33.15	26.70	55.83
Sufficient (MET ≥ 600/week)	40.76	14.45	22.10	45.93	26.82	36.40	29.59	60.73
**Smoking**								
No	42.17	15.50	23.27	47.12	27.20	36.55	28.08	59.48
Current	44.64	14.65	14.96	32.06	18.06	25.69	44.74	65.79
Former	57.25	27.03	26.75	44.87	20.13	25.42	32.43	56.76
**Water pipe (hookah)**								
No	42.77	15.94	23.29	46.50	26.16	35.15	29.06	59.59
Current	42.77	11.99	11.13	28.68	33.33	46.15	20.00	65.00
Former	47.69	16.70	18.89	42.11	31.71	40.62	33.33	55.56
**Family history BP**								
No	40.55	13.58	17.17	37.77	25.86	35.10	28.39	60.05
Yes	46.37	19.22	30.24	54.75	26.75	35.66	29.37	59.31
**History of DLP**								
No	37.94	12.40	20.67	44.91	22.24	31.32	27.99	61.95
Yes	47.71	19.18	24.34	46.45	29.13	37.96	29.45	58.40
**History of DM**								
No	39.74	13.16	17.85	40.15	26.61	35.72	31.38	61.21
Yes	60.67	30.98	41.02	59.76	25.98	34.93	24.78	56.93
**History of CVD**								
No	41.57	14.43	19.30	41.62	25.64	34.24	27.14	57.42
Yes	65.03	39.89	61.63	73.19	28.83	39.58	34.00	65.20

The economic status was defined based on the wealth index, which was calculated by multiple correspondence analysis (MCA) with household’s ownership of selected assets. Physical activity was estimated by metabolic equivalent of task (MET) category, which was calculated based on the international physical activity questionnaire (IPAQ) guideline. Dyslipidaemia was defined as either serum total cholesterol ≥ 200 mg/dL, high-density lipoprotein (HDL) cholesterol <35 mg/dL, or low-density lipoprotein (LDL) cholesterol ≥130 mg/dL or use of lipid-lowering medication. Diabetes Mellitus was defined as fasting plasma glucose levels (FBS) > 126 mg/dL, previously diagnosed DM, or current intake of antidiabetic agents. Cardiovascular diseases was defined if the participant reported any history of myocardial infarction, angiography, or any other related heart diseases.

*JNC8* eighth report of the Joint National Committee, *ACC/AHA* 2017 American College of Cardiology/American Heart Association, *BMI* body mass index, *MET* metabolic equivalent of task, *DLP* dyslipidemia, *DM* diabetes mellitus, *CVD* cardiovascular diseases, *Q* quartile

Among HTN-identified people, 45.85% were aware. About 35.42% of self-aware individuals received anti-HTN medication, which lowered the HTN to <90/140 mmHg in 59.63% of subjects. Nevertheless, after implementation of 2017 ACC/AHA, the rate of awareness, treatment, and control dropped to 22.72%, 26.37, and 28.94%, respectively (Table 1).

The most dominated changes in prevalence and awareness of HTN were observed among 45–54 years old, but young people less than 25 were more vulnerable in terms of treatment and control.

### Individual characteristics associated with HTN indicators according to the 2017 ACC/AHA guideline

Using 2017 ACC/AHA cut-off, the overall prevalence of HTN in Khuzestan province was 13,036 (42.85%), which changed to 39.4% after sex and age adjustment. In the studied population, the mean (±SD) age of hypertensive people was significantly higher than non-hypertensive people (P=0.000) and women were 41% less likely to manifest HTN compared to men (OR 0.59, 95% CI 0.56–0.62). Other independent protective factors were being Bakhtiary, getting married, having higher education level, doing more physical activity, and smoking, whereas being Arab, having high WHR and BMI, drinking alcohol, and having a family history of HTN were positively associated with prevalence of HTN (Table 2).

**Table 2:** Individual characteristics associated with prevalence, awareness, treatment, and control of hypertension according to the 2017 ACC/AHA guideline

Determinants of hypertension	Prevalence	Awareness	Treatment	Control
	OR (95% CI)^a^	OR (95% CI)^a^	OR (95% CI)^a^	OR (95% CI)^a^
**Age group**				
<25	1	1	1	1
25–34	1.09 (0.97–1.22)	1.44 (0.74–2.83)	0.83 (0.16–4.41)	1.45 (0.21–10.06)
35–44	**1.45 (1.29**–**1.62)**	**4.96 (2.63**–**9.48)**	1.07 (0.22–5.16)	0.41 )0.07–2.45)
45–54	**2.64 (2.36**–**2.96)**	**14.3 (15.2**–**53.9)**	1.4 (0.29–6.50)	0.31 (0.05–1.83)
55–64	**4.47 (3.98**–**5.03)**	**28.45 (15.2**–**53.9)**	1.74 (0.37–8.23)	0.24 )0.04–1.41)
=65	**7.29 (5.02**–**10.58)**	**51.2 (24.8**–**105.9)**	1.22 (0.23–6.14)	0.11 (0.01–1.05)
**Gender**				
Male	1	1	1	1
Female	**0.59 (0.56**–**0.62)**	**2.22 (2.01**–**2.46)**	1.16 (0.96–1.42)	1.22 (0.86–1.74)
**Marital status**				
Single	1	1	1	1
Married	**0.90 (0.83**–**0.98)**	**2.02 (1.49**–**2.76)**	1.14 (0.59–2.21)	0.73 (0.27–1.95)
Divorced	0.94 (0.74–1.2)	**1.85 (1.09**–**3.15)**	0.34 (0.09–1.32)	1.28 (0.14–12.12)
Widowed	1.11 (0.95–1.29)	**2.24 (1.59**–**3.19)**	1.33 (0.66–2.69)	0.58 (0.20**–**1.70)
**Socioeconomic status**				
Q1	1	1	1	1
Q2	1.00 (0.93**–**1.07)	1.06 (0.93**–**1.20)	0.97 (0.77**–**1.22)	1.22 (0.80**–**1.85)
Q3	**0.88 (0.83–0.95)**	1.07 (0.95**–**1.23)	1.03 (0.81**–**1.30)	1.16 (0.76**–**1.75)
Q4	**0.83 (0.77–0.89)**	1.11 (0.98**–**1.27)	0.89 (0.70**–**1.13)	1.37 (0.89**–**2.11)
**Education (year study)**				
Illiterate (<1)	1	1	1	1
Elementary school (1**–**5)	0.85 (0.79**–**0.92)	**0.84 (0.74–0.95)**	1.09 (0.88**–**1.35)	1.07 (0.72**–**1.60)
Guidance school (6**–**8)	0.74 (0.68**–**0.81)	1.04 (0.89**–**1.21)	1.09 (0.83**–**1.44)	1.59 (0.99**–**2.58)
High school (9**–**12)	0.73 (0.68**–**0.79)	**0.87 (0.76–0.99)**	**1.36 (1.06–1.74)**	1.34 (0.87**–**2.07)
Universities (>12)	0.66 (0.60**–**0.73)	1.00 (0.82**–**1.22)	**1.55 (1.06–2.25)**	1.44 (0.77**–**2.70)
**Ethnicity**				
Fars	1	1	1	1
Arab	**1.18 (1.10–1.26)**	1.10 (0.98**–**1.24)	**0.24 (0.19–0.30)**	**0.29 (0.19–0.46)**
Bakhtiary	**0.78 (0.72–0.84)**	1.07 (0.93**–**1.23)	**0.75 (0.59–0.96)**	0.71)0.48**–**1.04)
Lor	0.99 (0.90**–**1.09)	0.96 (0.81**–**1.15)	**1.53 (1.15–2.04)**	0.67 (0.44**–**1.02)
Others	**0.74 (0.56–0.99)**	0.94 (0.53**–**1.69)	**0.09 (0.01–0.66)**	Not enough
**BMI**				
Normal (18.6–25)	1	1	1	1
Underweight (≤18.5)	**0.65 (0.55–0.78)**	0.59 (0.31**–**1.14)	1.35 (0.39**–**4.73)	1.64 (0.34**–**7.90)
Overweight (25.1–30)	**1.46 (1.37–1.55)**	**1.57 (1.37–1.80)**	0.86 (0.66**–**1.11)	0.68 )0.45**–**1.04)
Obese (>30)	**2.15 (2.01–2.29)**	**2.09 (1.82–2.39)**	0.88 (0.68**–**1.14)	**0.49 (0.33–0.75)**
**Waist-hip ratio (WHR)**				
WHR<0.85	1	1	1	1
0.85≤ WHR <0.9	**1.23 (1.14–1.33)**	**1.24 (1.04–1.48)**	0.84 (0.59**–**1.19)	1.23 )0.70**–**2.17)
0.90≤ WHR <0.95	**1.43 (1.33–1.54)**	**1.44 (1.21–1.70)**	0.91 (0.66**–**1.24)	0.83 (0.49**–**1.39)
WHR ≥0.95	**1.58 (1.46–1.70)**	**1.75 (1.49–2.06)**	0.88 (0.65**–**1.18)	0.86 (0.53**–**1.42)
**Physical activity**				
Insufficient (MET<600/week)	1	1	1	1
Sufficient (MET ≥ 600/week)	**0.85 (0.80–0.91)**	**0.83 (0.74–0.92)**	1.12 (0.91**–**1.37)	0.98 (0.67**–**1.43)
**Smoking**				
No	1	1	1	1
Current	**0.80 (0.72–0.88)**	1.04 (0.85**–**1.28)	**0.58 (0.37–0.89)**	**2.51 (1.23–5.10)**
Former	0.96 (0.84**–**1.11)	1.21 (0.98**–**1.51)	0.66 (0.43**–**01.00)	1.67 )0.78**–**3.54)
**Water pipe (hookah)**				
No	1	1	1	1
Current	**1.15 (1.01–1.31)**	0.92 (0.66**–**1.26)	1.52 (0.85**–**2.70)	0.54 (0.17**–**1.68)
Former	**1.24 (1.02–1.51)**	1.25 (0.85**–**1.86)	1.19 (0.60**–**2.36)	1.12 (0.38**–**3.31)
**Family history BP**				
No	1	1	1	1
Yes	**1.35 (1.28–1.42)**	**2.37 (2.16–2.61)**	1.09 (0.92**–**1.29)	1.07 (0.79**–**1.45)
**History of DLP**				
No	1	1	1	
Yes	**1.10 (1.04–1.15)**	**0.90 (0.82–0.99)**	**1.41 (1.19–1.68)**	1.15 (0.84**–**1.58)
**History of DM**				
No	1	1	1	1
Yes	**1.50 (1.40–1.61)**	**2.04 (1.85–2.25)**	0.94 (0.79**–**1.11)	0.81 (0.59**–**1.10)
**History of CVD**				
No	1	1	1	1
Yes	**1.45 (1.30–1.63**)	**4.23 (3.66–4.88)**	1.16 (0.95**–**1.41)	**1.57 (1.13–2.20)**

The economic status was defined based on the wealth index, which was calculated by multiple correspondence analysis (MCA) with household’s ownership of selected assets. Physical activity was estimated by metabolic equivalent of task (MET) category, which was calculated based on the international physical activity questionnaire (IPAQ) guideline. Dyslipidemia was defined as either serum total cholesterol ≥ 200 mg/dL, high-density lipoprotein (HDL) cholesterol <35 mg/dL, or low-density lipoprotein (LDL) cholesterol ≥130 mg/dL or use of lipid-lowering medication. Diabetes Mellitus was defined as fasting plasma glucose levels (FBS) > 126 mg/dL, previously diagnosed DM, or current intake of antidiabetic agents. Cardiovascular diseases was defined if the participant reported any history of myocardial infarction, angiography, or any other related heart diseases. Bold values indicate statistical significance

*JNC8* eighth report of the Joint National Committee, *ACC/AHA* 2017 American College of Cardiology/American Heart Association, *BMI* body mass index, *MET* metabolic equivalent of task, *DLP* dyslipidemia, *DM* diabetes mellitus, *CVD* cardiovascular diseases, *OR* odds ratio, *CI* confidence interval, *Q* quartile

^a^Odds ratios were adjusted for age, gender, BMI, and physical activity

Older people (≥35 years old), females, not single individuals, and those with high level of BMI (>25 kg/m^2^) and WHR (≥0.85), a family history of HTN, history of DM, and CVD are more likely to be aware of HTN. Receiving HTN treatment was more prevalent in Lor people, those with history of DLP, and higher educated subjects (≥9 years). Individuals with history of CVD had 58% higher HTN control level after taking the HTN medication comparing to those with no CVD history (Table 2).

Although Arab people are 18% more likely to develop HTN comparing to Fars (OR 1.18, 95% CI 1.10–1.26), they were 76% less likely to receive HTN treatment (OR 0.24, 95% CI 0.19–0.30) and 71% less likely to have controlled HTN after receiving the anti-HTN drug (OR 0.29, 95% CI 0.19–0.46). Awareness was more prevalent in overweight, obese, and those with WHR>0.85. However, control of the treatment was 51% less prevalent in obese people (OR 0.49, 95% CI 0.33–0.75).

People with higher socioeconomic status had lower prevalence of HTN; however, there was no significant association between socioeconomic status and awareness, treatment, and control of the HTN (Table 2). Moreover, HTN was less prevalent in those with sufficient activity (OR 0.85, 95% CI 0.80–0.91) but the highly active people were less likely to be aware (OR 0.83, 95% CI 0.74–0.92) of HTN (Table 2).

In the nutrient assessment, we found no significant differences in the dietary habits of hypertensive and nonhypertensive group. In the sex stratification analysis, high fat intake was two-times more prevalent in hypertensive male population. In addition, a lower intake of salt and carbohydrate was observed in age group of 20–29 with HTN (data not shown).

## Discussion

After implementation of the 2017 ACC/AHA guideline, the prevalence of HTN in the Khuzestan province increased from 15.81 to 42.85% and the level of awareness (from 45.85 to 22.72%), treatment (from 35.42 to 26.37%), and control (from 59.63 to 28.94%) dramatically dropped. Since Khuzestan had the lowest level of healthcare development, and highest inequality of health services utilization in the country, a great need to expand the public health infrastructure for progressing towards the goals of UHC of HTN in this province is required.

In order to reduce the major adverse of CVD events, ACC/AHA has recommended a lower threshold of SBP and DBS (130/80 mm Hg) for diagnosis of HTN compared to what had been suggested earlier (140/90 mm Hg); however, there are debates about the proposed HTN cut-off and CVD benefit. Some studies reported reducing the SBP to 120 mm Hg level significantly decreased the risk of CVD [22], and a substantial reduction in risk was observed for levels SBP/DBP below 130–139 mm Hg [23, 24]. However, a recent systematic review and meta-analysis study found no reduction in risk of CVD-related death by reducing the cut-off from additional blood pressure lowering if the SBP at baseline was <140 mm Hg [25] and the authors further declared implementation of this guideline caused a significant increase in the proportion of adult diagnosed with HTN, with no noticeable effect on the reducing CVD morbidly and mortality [26]. Here, we found the number of hypertensive patients in Khuzestan province increased from 4809 (15.81%) to 13,036 (42.85%). A lower changes have been documented in other countries like China (>18 years) [27], USA (>20 years) [28], and Sweden (25–74 years) [26] in comparison with the previous report from Iran (>25 years) [6], which indicates the number of people with SBP 130–140 or DBP 80–90 was higher in Iran and our country would gain greater benefit of reduction in CVD mortality in the future if the 2017 ACC/AHA guideline is implemented.

**Fig. 1:**
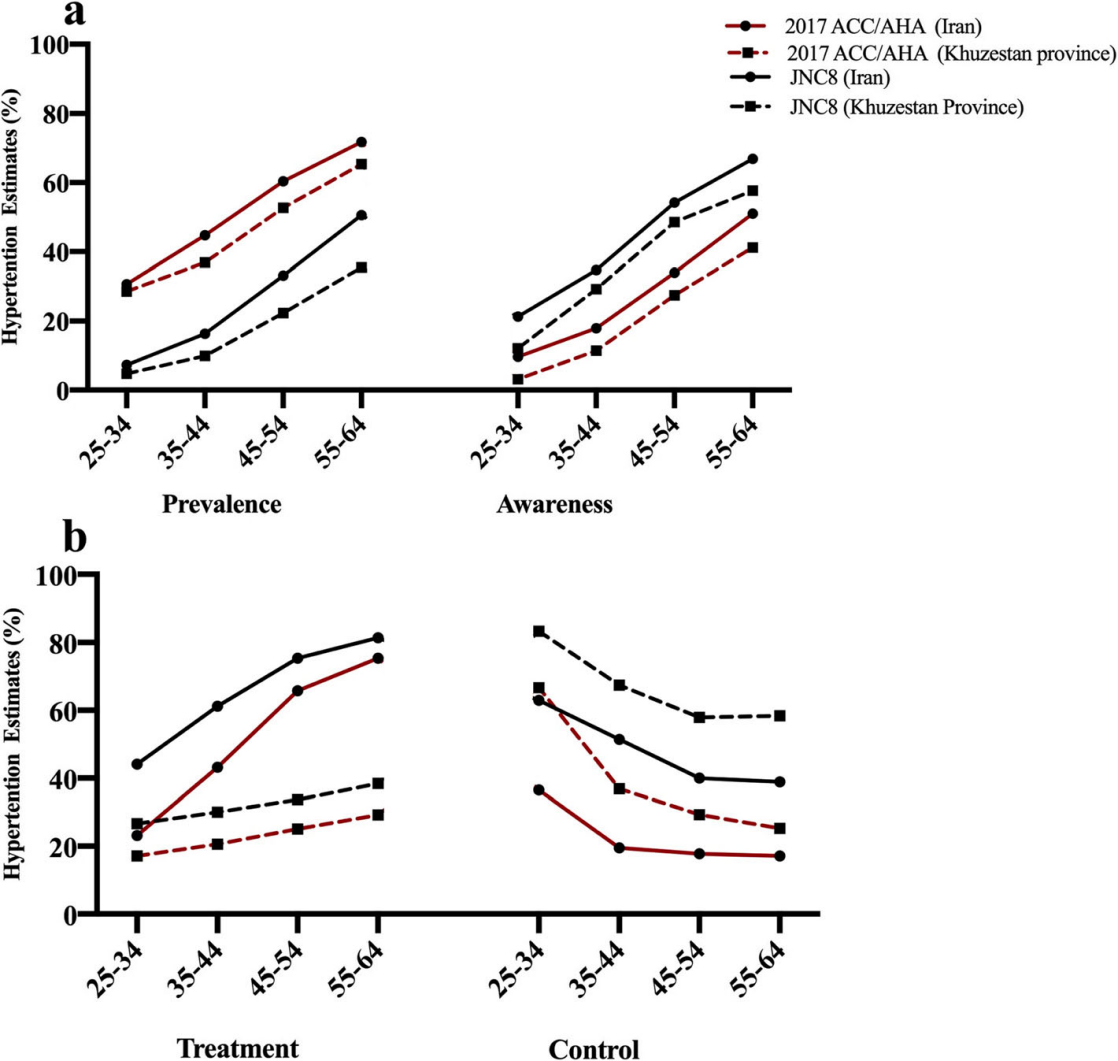
The impact of 2017ACC/AHA and JNC8 guidelines on the prevalence, awareness (**a**), treatment, and control (**b**) of hypertension in different age groups in Khuzestan province, comparing to the national average rates of prevalence, awareness, treatment, and control of hypertension

The increased prevalence of HTN was observed in all age group of Khuzestan population: lowest in under 25 years of age (from 3.36 to 24.44%) and highest in 45–54 years old individuals (from 22.28 to 52.73%). Although Khuzestan has critical health issues, the overall prevalence of HTN in this province is lower than the estimated HTN prevalence in Iran [6] (Fig. 1a) and the effect of new guideline on older population of Khuzestan was more dominated than overall population of the country. Moreover, a noticeable shift of HTN prevalence has been observed among male population. Although almost equal number of male and female were diagnosed with HTN previously (17.79% vs 14.70%), we observed a more significant increase in HTN among male after applying the 2017 guideline (50.30% vs 38.70%).

According to the JNC8 report, we found that 45.85% of the hypertensive participants in Khuzestan province were aware of HTN, which is lower than the overall awareness estimates in the country [6] (Fig. 1a). Khuzestan has the lowest level of healthcare development within country [13], which required investment of more efforts to improve the health education in the community. After adopting the 2017 ACC/AHA, the awareness rate dropped to 22.72%, which was more noticeable in the 45–54 age group. Since 52.73% of this age group are now classified under the HTN category, a substantial call for action to raise public awareness is warranted.

Moreover, the HTN treatment strategy has been revised in the 2017 ACC/AHA guideline and those with SBP 130–139 or DBP of 80–89 were also recommended to received anti-HTN treatment if they are +65 years older, or the clinical CVD or 10-year atherosclerotic cardiovascular disease risk is 10% or greater [7]. Following this guideline, many high-risk adults might be protected against the development of HTN-related chronic diseases. However, the number of people who need anti-HTN treatment has been expected to increase from 8.1 million to 15.6 million in the USA and from 74.5 million to 129.8 million in China [8], which may impose a considerable impact on the health system in these countries. In this study, 4738 (15.53%) of total sample were eligible to receive HTN therapy based on JNC8. After ACC/AHA, additional 1724 subjects with SBP 130–139 or DBP of 80–89 and history of CVD or DM become candidate for pharmacological interventions, which increase the treatment demand to 21.18%. However, our study was limited to people aged 65 and younger and estimating the real impact of this guideline on treatment eligibility in our population was not feasible. Another recent study conducted in Iran estimated that the number of adults who met the criteria for treatment with anti- hypertensive medication would increase from 13.4 million to 17.2 million and the annual cost of pharmacologic therapy would change from $510 million to $653 million [6]. Although the increased prevalence of HTN was more noticeable in Iran, the treatment burden in this country was lower compared to China and the USA, which might be due to smaller population of 65+ years old in Iran. Therefore, the impact of treatment costs on the health system under the 2017 ACC/AHA guideline slightly increased.

However, in Khuzestan province, the effect of new guideline would be more noticeable. For example, based on JNC8 report, an average of 81.4% of self-aware individuals between 55 and 64 years old received HTN treatment at the national level [6] but this level among the same age group in Khuzestan was 38.43% (Fig. 1b), which indicates a serious lack of HTN treatment among hypertensive people in this province. The inequality of health services utilization in this province is the main cause of lower treatment rate in this province. This inequality was also observed among different ethnicity groups within the province. Although Arab people are 18% more likely to develop HTN comparing to Fars (OR 1.18, 95% CI 1.10–1.26), they were 76% less likely to receive HTN treatment and 71% less likely to have controlled HTN after receiving the anti-HTN drug. HTN treatment also was 25% less prevalent in Bakhtiary people. Although, Khuzestan is most industrialized province in the country, it is the most deprived province in the country in term of healthcare. Therefore, implementation of the 2017 ACC/AHA guideline may considerably delay the progression towards UHC of HTN in this province.

Among treated individuals, 59.63% had blood pressure <130/80 mmHg. Under ACC/AHA guideline, the blood pressure <130/80 mm Hg was considered as the target cut-off; therefore, the control rate dropped to 28.94%, which highlight a great need to expand the public health infrastructure for further managing the substantial increase in the public health burden of HTN. Although the treatment rate in Khuzestan was much lower than mean percentage of the country, the controlled level was higher in this province (Fig. 1b).

Considering the 2017 ACC/AHA guideline, 6574 participants younger than 65 years old have been additionally diagnosed with HTN, who might benefit from earlier interventions. Apart from high-risk individuals, this group of people are not eligible for pharmacological interventions; therefore, modification of life style and nutrient diet is critical to reduce the risk of HTN-related disorders in the future. For example, 22.56% of obese precipitants were diagnosed with HTN previously, and this value jumped to 53%. Although HTN was 15% less prevalent in those with sufficient activity, they were 17% less likely to be aware of HTN. In this study, the mean of sodium intake was estimated to be 8.74 g/day, which exceeds the current WHO recommendations (2 g/day (d) of sodium, equivalent to 5 g/d of salt) [29]. Based on 2017 ACC/AHA, we estimated about 79.38% of hypertensive people consume salt over the threshold, 75%.82 need to lose extra weight, and 22.48% are required to do more physical activity.

Our study has certain limitations. This is a cross-sectional based study, and a causal relationship cannot be inferred. Moreover, based on the HTN 2017 ACC/AHA, those with SBP 130–139 or DBP of 80–89 were also recommended to receive anti-hypertension treatment if they are +65 years older. However, our study was limited to people aged 65 and younger, and further study covering all range group is required to estimate the real impact of treatment after implementation of new guideline.

## Conclusion

In the ACC/AHA guideline, a higher number of individuals with the pre-hypertension condition had been shifted into the hypertension category, and the level of awareness, treatment, and control was dramatically fallen, which highlight a great need to expand the public health infrastructure for further managing the substantial increase in the public health burden of hypertension.

## Data Availability

The datasets from the current study are included within the article.

## Abbreviations

HTN: Hypertension
CVD: Cardiovascular diseases
JNC8: Eighth report of the joint national committee
SBP: Systolic blood pressure
DBP: Diastolic blood pressure
ACC: American College of Cardiology
AHA: American Heart Association
KCHS: Khuzestan Comprehensive Health Study
FBS: Fasting plasma glucose levels
BMI: Body mass index
WHR: Waist-to-hip ratio
MCA: Multiple correspondence analysis
MET: Metabolic equivalent of task
IPAQ: International physical activity questionnaire
HDL: High-density lipoproteins cholesterol
FFQ: Food frequency questionnaire
SD: Standard deviation
