# Correction to: Impact of 2017 ACC/AHA guideline on prevalence, awareness, treatment, control, and determinants of hypertension: a population-based cross-sectional study in southwest of Iran

**DOI:** 10.1186/s12963-021-00272-1

**Published:** 2021-10-19

**Authors:** Fatemeh Sadeghi, Bahman Cheraghian, Zahra Mohammadi, Sadaf G. Sepanlou, Sahar Masoudi, Zahra Rahimi, Leila Danehchin, Yousef Paridar, Farhad Abolnezhadian, Mohammad Noori, Seyed Ali Mard, Ali Akbar Shayesteh, Hossein Poustchi

**Affiliations:** 1 Department of Global Public Health, Karolinska Institute, Stockholm, Sweden; 2 Alimentary Tract Research Center, Imam Khomeini Hospital Clinical Research Development Unit, Department of Biostatistics and Epidemiology, School of Public Health, Ahvaz Jundishapur University of Medical Sciences, Ahvaz, Iran; 3 Liver and Pancreatobiliary Diseases Research Center, Digestive Diseases Research Institute, Tehran University of Medical Sciences, Tehran, Iran; 4 Digestive Disease Research Center, Digestive Disease Research Institute, Tehran University of Medical Sciences, Tehran, Iran; 5 Hearing Research Center, Department of Biostatistics and Epidemiology, School of Public Health, Ahvaz Jundishapur University of Medical Sciences, Ahvaz, Iran; 6 Behbahan Faculty of Medical Sciences, Behbahan, Iran; 7 School of Medicine, Dezful University of Medical Sciences, Dezful, Iran; 8 Shoshtar Faculty of Medical Sciences, Shoshtar, Iran; 9 Ahvaz Jundishapur University of Medical Sciences, Ahvaz, Iran; 10 Abadan Faculty of Medical Sciences, Abadan, Iran; 11 Clinical Research Development Unit, School of Medicine, Alimentary Tract Research Center, Imam Khomeini Hospital, Ahvaz Jundishapur University of Medical Sciences, Ahvaz, Iran

## Correction to: Population Health Metrics (2021) 19:26. 10.1186/s12963-021-00260-5

Following the publication of the original article [1], the authors reported a missing grant number in their Funding declaration. The updated Funding declaration is as follows:

The project was funded by the National Institute for Medical Research Development (NIMAD, Grant number: 940406).

The original article has been corrected to include this Funding declaration.
